# Detection dogs fighting transnational narcotraffic: performance and challenges under real customs scenario in Brazil

**DOI:** 10.3389/fvets.2024.1380415

**Published:** 2024-05-16

**Authors:** Gustavo Machado Jantorno, Carlos Henrique Xavier, Marcelo Eduardo Peixoto Magalhães, Márcio Botelho de Castro, Concepta McManus, Cristiano Barros de Melo

**Affiliations:** ^1^Graduate Program in Animal Sciences (PPGCA/FAV), University of Brasilia (UnB), Brasilia, Brazil; ^2^Center for Detection Dogs (CNK9), Customs/Aduana, Vitória, Espirito Santo, Brazil; ^3^Head of the Center for Detection Dogs (CNK9), Customs/Aduana, Vitória, Espirito Santo, Brazil

**Keywords:** border, crime prevention, sniffer dogs, drug trafficking, narcotic detection

## Abstract

Narcotic Detection Dogs (NDDs) are essential tools in the fight against drug trafficking, acting with high precision and improving efficiency at border posts. When trained efficiently, these dogs can detect a great variety of compounds, such as cocaine, marijuana and its derivatives, and synthetic drugs, among others. Most of the knowledge on canine detection processes and efficiency has been determined in experimentally controlled conditions, but narcotic seizures detected by dogs in realistic anti-drug operations have not yet been critically determined in a Country with continental dimensions such as Brazil. This study aimed to evaluate the data set concerning the performance, operations, efficiency, and success rate of NDDs used by the Brazilian Customs Authority (Aduana) in the fight against drug trafficking. Narcotic seizure rates increased in luggage and packages detected by NDDs working at border crossings from 2010 to 2020, with an estimated value of over US$ 2 billion in losses to the cocaine drug trafficking business. NDD units also increased most narcotic groups seized in the same period. The number of NDDs and anti-drug operations, and Customs Border Post (CBP) influenced the rates of drugs seized. NDDs provided an increase of 3,157 kg/animal of drugs seized for every new dog introduced into the inspection systems.

## 1 Introduction

The high impact of illicit drugs on Societies worldwide requires hard work in the fight against drug trafficking, including scanners, video monitoring, intelligence services, and Narcotic Detection Dogs (NDDs) with high olfactory power and accessible training capacities (1) for Customs and border inspection (2, 3). The danger and price linked to narcotrafficking for the security of the population and countries justify investments in NDD units (4). Drug consumption and trafficking cause severe adverse effects on Societies, especially on the young population, promoting psychological disorders, schizophrenia, and mental sicknesses such as observed in people with daily use of highly potent marijuana (5, 6).

Drug trafficking is a highly profitable business with, in general, a strong political and economic influence, frequently involving armed conflicts, violence against citizens, and the involvement of other illegal organizations. It also involves actions against Police and Drug Trafficking Control Systems and attempts to maintain control over the drug trafficking routes, as evidenced in Colombia, Mexico, and Brazil (7). Cocaine trafficking is the most violent business associated with the illicit drug trade, which generally includes the corruption of government agents (8). The highly organized crime of cocaine trafficking operates in a sizeable redundant network with transient links, reorganization of trafficking routes, and drug suppliers to avoid seizures by Customs authorities (8, 9).

Dogs detect odors in the most diverse environments and are used to search for several substances, products, animals, and even man worldwide without direct contact (3). These dogs, with their exceptional sense of smell, combined with learning and conditioning training, can find hidden targets such as explosives, illicit drugs, and missing persons, as well as detect diseases and the need for certain medications by persons (1, 2, 10). The effectiveness of dogs as detectors has been proposed as a result of their olfactory anatomy (neurons, olfactory receptors, and olfactory bulb) (11) and their behavior in searching for the desired information (12).

Narcotic Detection Dogs can be trained to detect drugs such as synthetic cathinone (amphetamine) and have shown that they can be quickly prepared to detect new threats (ethylene and α-PVP amphetamine) in a matter of weeks (13). NDDs are considered highly accurate, low-cost within their economic context compared to other police–border protection priorities, and reliable real-time narcotics detection tools, increasing the efficiency and speed of inspection at customs posts and frontiers (14–16).

Experimentally, narcotics detection is influenced by the persistence of residual odors, increasing the difficulty for NDDs searching for marijuana, hashish, amphetamine, cocaine, and heroin (17). On the other hand, studies on narcotics seizure by NDD units in realistic operations against drug trafficking in Customs Border Posts (CBPs) are still lacking, mainly due to the difficulty in accessing sensitive data, as well as the risks involved in fighting against international crime organizations. The Brazilian Customs Service (Aduana) has invested in NDDs, including the training and maintaining high-performance working dogs to fight against drug trafficking (18, 19).

Thus, the present study evaluated the performance, efficiency, and the effect of the number of anti-drug operations on the success rate of drug detection by NDDs, which includes year, month, number of anti-drug operations, locality of drug apprehensions, seizure rates of specific drug types, rates of drug detection by individual NDDs and drug seizures by CBPs in a real operating scenario.

## 2 Materials and methods

### 2.1 Sample, data, and type of narcotics seized

We analyzed data on narcotics detection by dogs from the Brazilian Customs Service between 2010 and 2020. The use of NDDs in one anti-drug inspection operation included the routine inspection of luggage, containers, cars, ships, planes, etc., or in the form of a surprise check, in which the inspection or repression of drug trafficking is carried out without prior warning. Each anti-drug operation involved different locations (CBPs), variable environments, and specific and intrinsic conditions required against drug trafficking, routinely implemented or based on sensitive information provided by Brazilian Anti-Drugs Intelligence Organizations.

Drug seizures made by NDDs from the Brazilian Customs anti-drug units inspecting baggage, packages, and cargo were evaluated (18, 19) by year, type of drug, weight (or the number of pills for ecstasy), number of NDDs, number of seizures, and location of the CBPs. Seizures were grouped by narcotics types as tetrahydrocannabinol (THC) based products: marijuana (plant material from the cannabis plant), hashish^1^ (compressed resin of flowers of the cannabis plant extracted trichomes, with higher levels of THC), and Skunk^2^ (from the non-pollinated cannabis plant with higher levels of THC than hashish, 15 vs. 5%); other plant-based products: cocaine (from coca plant—C_17_H_21_NO_4_), crack (free base form of cocaine), pasta (a by-product of processing raw coca leaf into cocaine) and heroin (crude preparation of diamorphine from opium); and Synthetics: lysergic acid diethylamide (LSD), meth (methamphetamine), MDMA crystals (3,4-methylenedioxy-methamphetamine), and ecstasy (tablets).

In this study, we exclusively used an aggregated data set and information collected in a real scenario of anti-drug operations on drug seizures, detection dogs, and Customs Border Posts. The Brazilian Customs provided all aggregated data sets, and their use was explicitly authorized. Brazilian Customs owned all evaluated detection dogs, which were not submitted to any experimental procedures or protocols previously outlined for this study. According to Brazilian laws, the approval of this study by an Ethics Committee for Animal Experimentation is not applicable. The data set on anti-drug operations supplied by the Receita Federal of Brazil included the identification of the use of each NDD in each anti-drug operation but not the individual dog’s work cycle times (time searching/resting). All procedures, training, resting periods, and keeping detection dogs in Brazilian Customs followed the Animal Health and Welfare Act Number 15, 2013, revised and updated to January 22, 2022.

### 2.2 NDDs individual performance

From 2010 to 2020, we evaluated all seizures and types of illegal drugs intercepted by NDDs. These animals were German Shepherd and Belgian Malinois dogs, adults, males, and females, and only a few were neutered for reasons related to management or veterinary advice. New dogs were added after the selection and training process (16). Additionally, we analyzed the individual performance of 27 NDDs employed in 17 different Customs Border Posts (CBPs) in a real scenario from January to June 2021. The identity of NDDs was omitted for security reasons, and they were referred to as K9-1 to K9-27.

### 2.3 Narcotics interception in customs border posts

We assessed data set on operations for narcotics interception performed with NDDs in 17 Customs Border Posts (CBPs). CBPs were located at Brazil’s airports, ports, land borders, and international postal reception centers. The identification and location of CBPs were also omitted and nominated as CBP-1 to CBP-17 for security and to preserve strategic information against drug trafficking crime.

### 2.4 Data analysis

Different models were tested (linear, quadratic, and cubic), and the one with the highest *R*^2^ was selected as long as it was significant. Regression analyses were used to identify the influence of year on drug types seized. Multivariate data analysis included correspondence (year and substance seizure), cluster (by year and drugs), principal component, regression, and logistic analyses. The individual performance of the NDDs active in inspections between January and June 2021 was also determined, considering variables such as the weight of drug seizures per dog, the number of counter-narcotics operations each participated in, and the success rate of drug seizures. The Proc General Linear Model (GLM) was used to evaluate the effect of the month, the CBPs, the interaction between the NDDs and the number of operations, and the interaction between the number of operations, the number of seizures, and the success rate of drug seizures.

Tukey test (*p* < 0.05) compared the number of operations (number of monthly anti-drug operations employing NDDs) with drug seizures (number of operations with drugs seized). A logistic regression assessed the success rate of drug detections (NDDs drugs detection—0/1—no/yes). All these data analyses were performed with SAS v.9.4 software (20).

### 2.5 Special permissions

All data used in this study are under special authorization by the Brazilian Customs (Aduana) through Secretaria da Receita Federal do Brazil (RFB—Brazilian Revenue Service) and permits from the Customs Inspection and Repression Coordination (COFIR-RFOC/SRF) and CNK9 Administration (NDDs National Center) on March 7, 2017. The permit was renewed on March 6, 2023, by the RFB’s General Coordination for Combating Smuggling and Embezzlement Practices (COREP/Brazil).

## 3 Results

Brazilian Customs seized 97,788 kg of marijuana from 2010 to 2020, and NDDs accounted for 12.7% (12,424 kg) of seizures of this drug. From a total of 179,380 kg of cocaine seized in the CBPs, 37% (66,419 kg) were apprehended by NDD units. The annual amount of narcotics (Kg) detected by NDDs from the Brazilian Customs is shown in Supplementary Table 1.

**Figure 1:**
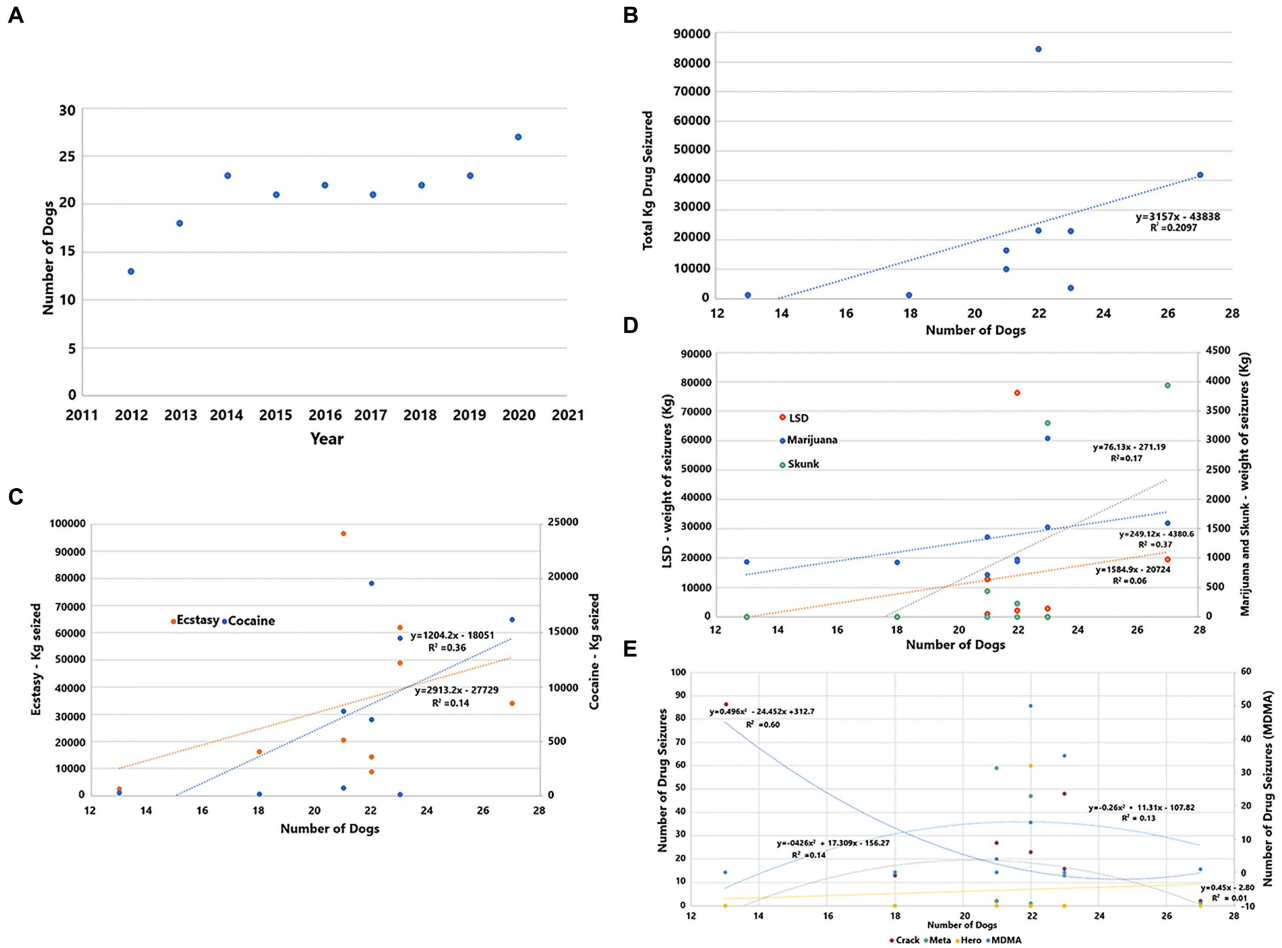
**(A)** Number of dogs operating by year; **(B)** Total of drug seizures (kg) by the total number of dogs; **(C)** Ecstasy and cocaine seized (kg); **(D)** Seizures (kg) of LSD, marijuana, and skunk; and **(E)** Crack, meth, heroin, and MDMA seizures (kg).

The number of NDDs in anti-drug operations increased from 2012 to 2014, thus remained stable until 2019, and rose again in the following years (Figure 1A). Raising the employment of NDDs in anti-drug operations increased the total weight of drugs seized by dogs to 3,157 kg/animal, which are the totals shown in Figure 1B. There is more than one point, as each point refers to a certain year, such as observed with 21 dogs used in 2015 and 2017 and 23 dogs in 2014 and 2019. Seizures of Ecstasy (Figure 1C) were excluded from the total weight (kg) of drugs seized and accounted for by the number of pills. Seizures of LSD (1,585 blotters), marijuana (76 kg), skunk (250 kg), ecstasy (2,913 tablets), and cocaine (1,200 kg) were increased (Figure 1D) with the addition of more NDDs in CBPs. In contrast, crack seizures decreased during this study, and even with the increment of NDDs in anti-drug operations (Figure 1E), there was no significant effect on crack, meth, heroin, and MDMA seizures.

**Figure 2:**
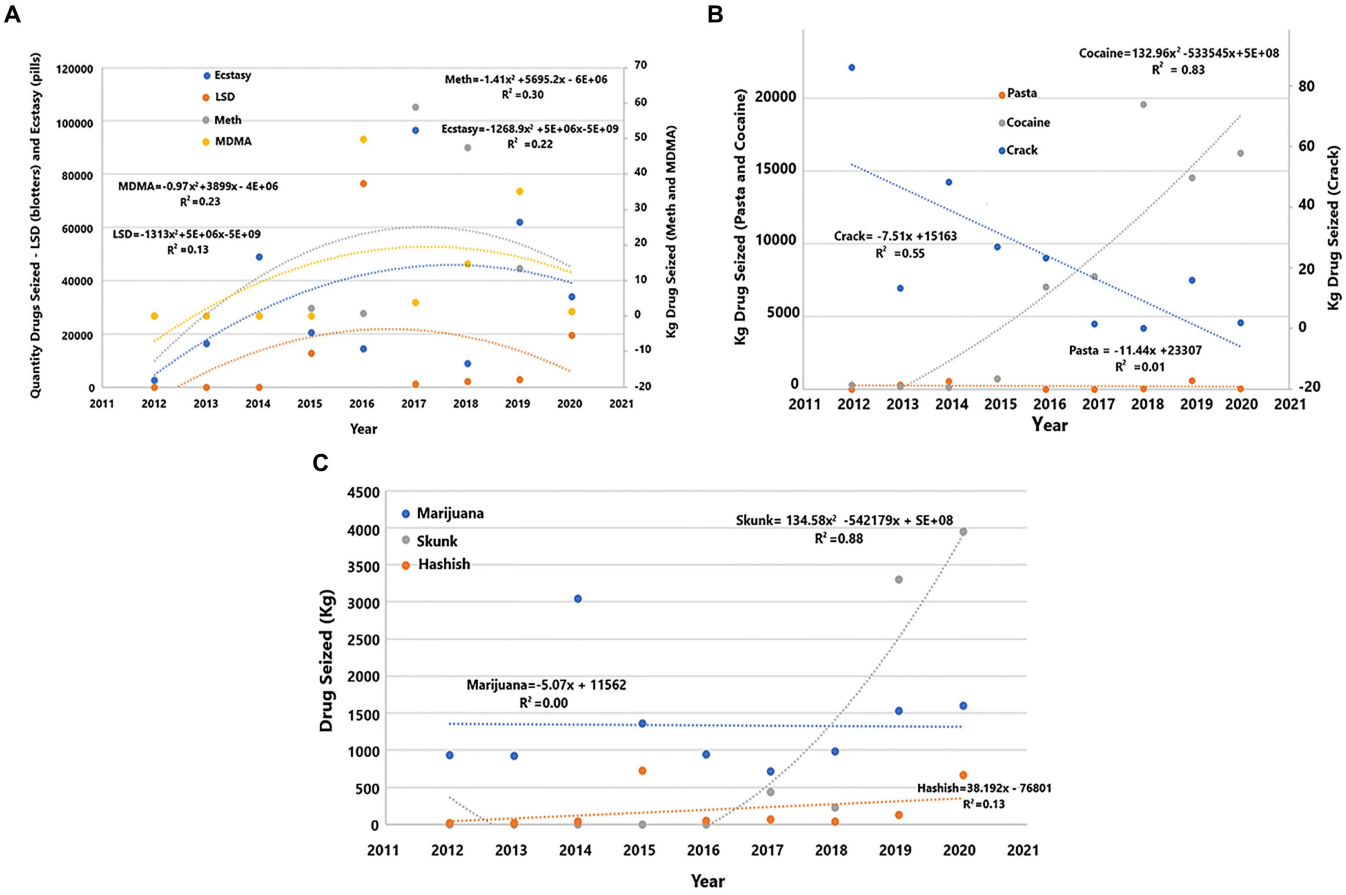
Regression analysis showing seizures by narcotic groups related to NDDs employed at CBPs in Brazil from 2012 to 2020. **(A)** Ecstasy, LSD, meta, and MDMA; **(B)** Pasta, cocaine, and crack; **(C)** Marijuana, Skunk, and hashish. Hero, Heroin; MDMA, 3,4-Methyl enedioxy methamphetamine; Meth, Methamphetamine; Pasta, Basic cocaine paste; LSD, Lysergic acid diethylamide.

The regression analysis (linear e quadratic) of the seizures by narcotics groups in Brazil from 2012 to 2020 confirmed an increase in seizures of synthetic drugs (ecstasy, LSD, meth, and MDMA) (Figure 2A) in 2014 and 2016, growth in cocaine and decrease in crack apprehensions between 2013 and 2020 (Figure 2B), and an increase in skunk seizures from 2016 to 2020 (Figure 2C) related to NDDs working in CBPs. A cluster analysis of years and drug seizures by NDDs showed little impact of NDDs on the number of overall narcotics seizures in 2010, 2011, and 2012 (Supplementary Figure 1).

Correspondence analysis between the years (2012 and 2020) and narcotic-type seizures in anti-drug operations employing NDDs showed a gradual increase in narcotics seizures since 2013 (Supplementary Figure 2), except for heroin, which was removed from this analysis due to the low number of seizures. From 2012 to 2017 (except 2016), marijuana, ecstasy, crack, and pasta were the most frequent narcotic types seized. There was a close correspondence to the seizures of LSD in 2016, cocaine and meth in 2018, and MDMA, skunk, and hashish in 2019 and 2020.

**Figure 3:**
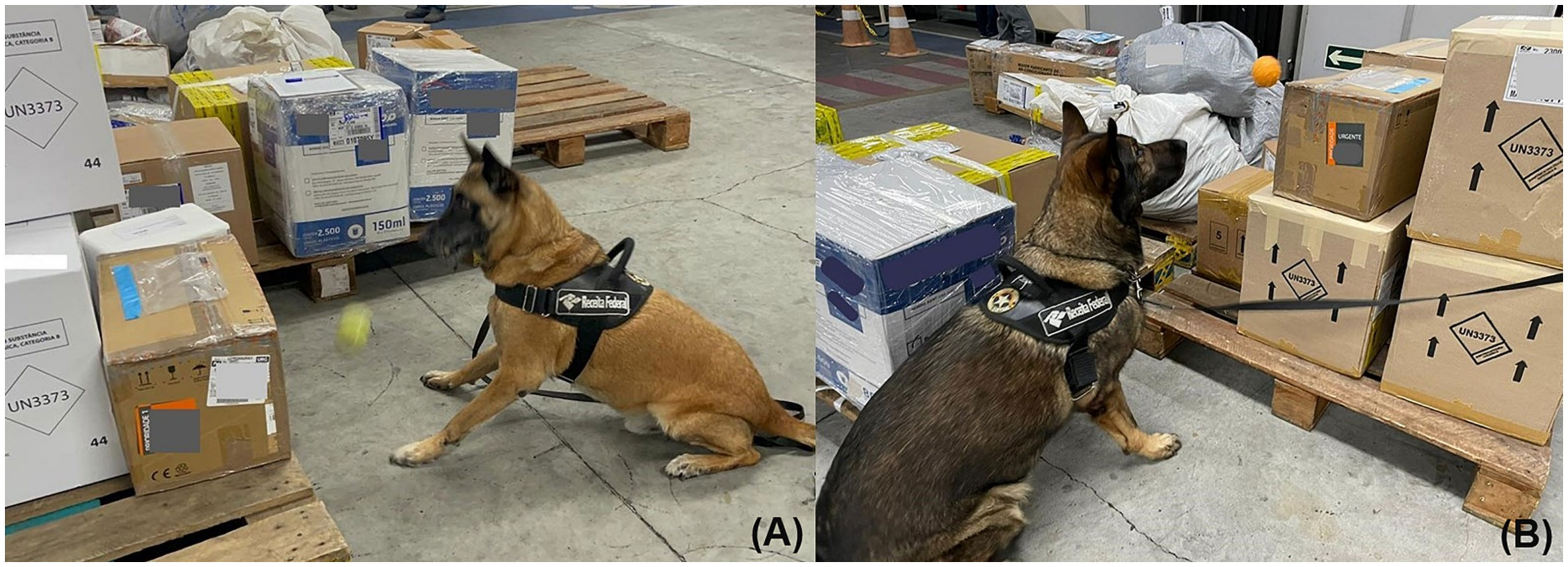
Two NDDs—**(A)** K9-24 and **(B)** K9-23, respectively, searching for illicit drugs at CBP. NDDs have been rewarded with a ball after assertively indicating the presence of narcotics inside packages.

**Figure 4:**
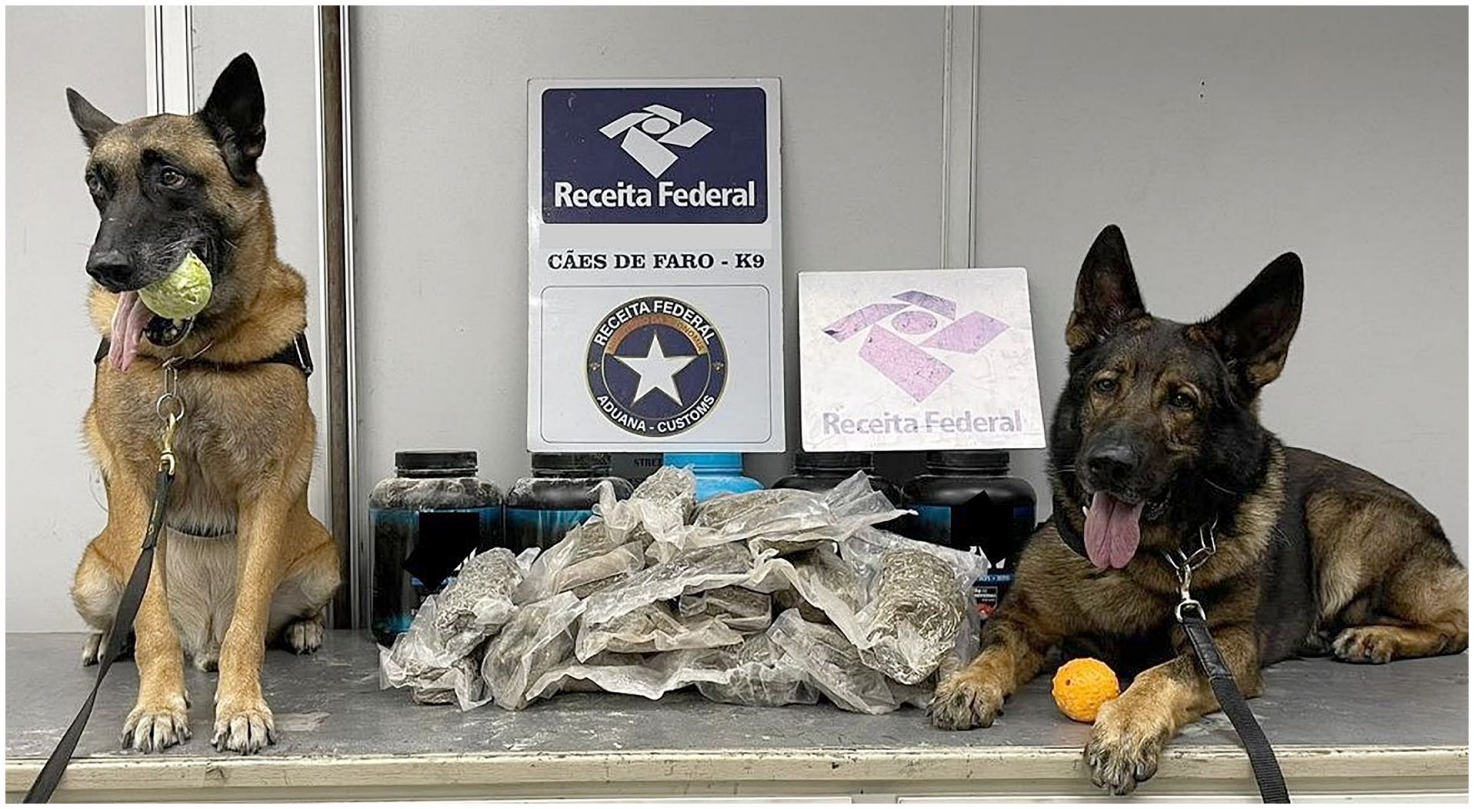
NDDs K9-24 and K9-23, respectively, with Cannabis derivates detected and seized (packages) that were going to be shipped within luggage to other countries in an international air flight.

Between January and June 2021, of 1,544 anti-drug operations employing 27 NDDs, 305 resulted in drug seizures (19.75%). The polynomial regression analysis of the number of anti-drug operations using NDDs and the seizure rate for narcotics detection evidenced an increase in the number of seizures up to 30 anti-drug consecutive operations using NNDs. Above 30 anti-drug consecutive operations using an NND, a decrease in the dogs’ performance and a marked reduction of narcotic apprehensions in the following operations (Supplementary Figure 3) were observed. The individual performance of an NDD and the number of operations influenced the amount of illicit drugs seized (*p* < 0.05—Supplementary Table 2) and also the success rate in drug detection (*p* < 0.05—Supplementary Table 3), which significantly varies between NDDs, with the best results achieved by K9-19, K9-23, and K9-24 (Figures 3, 4). The number of operations and the CBPs influenced seizures of narcotics by NDDs (*p* < 0.05), with higher success rates in CBP-3, CBP-5, and CBP-7 (Supplementary Table 4). The success rate of anti-drug operations in the seizure of narcotics was influenced by the CBPs (*p* < 0.05), remarkably in CBP-5, CBP-7, and CBP-9 (Supplementary Table 5).

## 4 Discussion

The global trade of narcotics and illicit drugs is a billionaire business mainly controlled by international trafficking organizations with high impacts on Societies and Countries. Efficient tools against drug trafficking require enormous efforts to monitor borders and Customs, hindering, interrupting, and curbing illegal transport and trade of illicit drugs. In a real scenario, this study demonstrates the employment of NDD units from Brazilian Customs in anti-drug operations from 2010 to 2020.

Cocaine and cocaine-derivated compounds were the most frequent drugs intercepted by NDDs in anti-drug operations in CBPs in this study, representing over one-third of all cocaine apprehensions in the period, followed by THC-containing drugs. From 2014 to 2019, there was an increase in the number of NDDs in anti-drug operations, which resulted in a growing number of drugs seized (Supplementary Table 1). One of the most remarkable findings observed in this study was every dog working in drugs detection at CBPs in this period significantly provided an increase in LSD (1,585 blotters), marijuana (76 kg), skunk (250 kg), ecstasy (2,913 tablets), as well as cocaine (1,200 kg) seizures (a total average of 3,157 kg of drugs detected by every single NDD), but did not affect the amount of crack, meth, heroin, and MDMA seized. Skunk seizures considerably increased, and marijuana and hashish showed minor enhancements in the period. Similarly observed herein, NDDs accounted for 15% of total narcotic seizures in Iran in 2014, representing around 1,950 kg of heroin with a market price of approximately US$ 780 million (21).

Brazil is considered a Country with significant transit and destination for cocaine and cocaine-derivative products, with an expressive internal domestic drug consumption problem and a vast border with sources of the drugs such as Bolivia, Colombia, and Peru (22). Considering prices of around US$ 29,000 per kg of cocaine in the United States in 2019 (23, 24), apprehensions of cocaine (66,419 kg) related to NDDs in Brasilian CBPs in the period we evaluated represented US$ 1,926,151,000 in losses for the drug trafficking. Marijuana and derivates have cannabidiol (CBD) and delta-9-tetrahydrocannabinol (THC) as the two major cannabinoid compounds (25) and represented a significant amount of drug apprehensions in this study. These plant substances and derivates have been the most consumed illicit drugs worldwide, with over 200 million users in 2021 (24), which represents almost 4% of the global population (26).

Regarding the high added value and use by higher social classes, cocaine is one of the most trafficked and seized drugs worldwide. In opposition, crack stones derived from cocaine are usually consumed by lower social classes and sold in the streets at a low cost (27). In general, crack is a low-cost drug manufactured from unrefined cocaine in clandestine laboratories within the country itself to be sold on the streets, being not economically advantageous for trafficking compared to refined cocaine, which can explain lower apprehensions of crack compared to cocaine, related to NDDs employment in CBPs. Even with the employment of NDDs trained to detect crack in addition to the use of other tools (e.g., scanners) at border crossings (CBPs), seizures of crack are usually inexpressive, our findings suggest a low rate of trafficking of this drug in these locations. Therefore, even considering several factors influencing the rate of illicit drug apprehensions, such as different trafficking routes and ways for different drug types, trade demand, and others, cocaine and derivates and marijuana and THC-related products are expected to be the most trafficked drugs and detected by NDDs, as observed in this study.

Our study also showed an increase in seizures of synthetic drugs (such as ecstasy, LSD, meth, and MDMA—Supplementary Table 1) between 2014 and 2016 in the CBPs evaluated. NDDs are usually involved in the seizure of synthetic drugs, such as LSD and methamphetamines, at airports and the post office. Continually new synthetic drugs are made by altering the molecular structures of controlled or illegal substances and mainly represent synthetic cannabinoids and synthetic cathinone, which usually have severe health consequences for users and have been popularized for recreational use (28). Illegal synthetic drug consumption and trade have also experienced a significant increase in South America, with around 130 new synthetic psychotropic substances identified in the region from 2013 to 2017 (29). In Europe, a similar ascension in the consumption and trade of synthetic drugs has been detected, with the Netherlands being one of the most significant producers (methamphetamines, amphetamines, and MDMA) (30).

Due to the high dynamics of new synthetic drug production, a constant update in the odourants must be used in NDD training periodically to keep a high performance of dogs, according to new synthetic drugs seized (16). Results observed in synthetic drugs intercepted by NDDS in Brazil possibly reflect the increase in the production, illegal trade, and demand for these substances in South America. In addition, Brazil hosted significant worldwide events, such as the World Soccer Cup in 2014 (around 1,736,645 international visitors) (31) and the Olympics in 2016, with a large flow of foreign and Brazilian citizens during this period, which could influence an increased demand for these drugs and apprehensions by NDDs.

From 2010, 2011, to 2012, NDDs had an unremarkable impact on illicit drug seizures (Supplementary Table 1). At the beginning of NDDs deployment in anti-drug operations, there were few trained NDDs for drug detection in CBPs, which probably resulted in a low impact on drug apprehensions in these years. Since 2013, anti-drug operations employing NDDs showed an onward increase in narcotics seizures, except for heroin, which may be justified considering Brazil is not a traditional heroin route (32) and has an insignificant domestic consumption. Experimentally, heroin usually has a low rate of detection by NDDs (17). Furthermore, changes in the demand for different drugs, repression in the drug trade, and the trafficking routes (33) might also influence narcotic detection by NDDs in this study. New NDD teams were introduced in the Brazilian Customs in 2013, and improvements in the training process of dogs employed in anti-drug operations possibly impacted the quantity of narcotics seizures at CBPs in the subsequent years (16).

Another remarkable observation in this study was the performance of 27 NDDs in 1,544 anti-drug operations for 6 months in 2021 (Supplementary Table 2), evidenced a drop in the performance of NDDs in drug detection and a decrease in apprehensions after 30 consecutive operations (Supplementary Figure 3). Even considering the limitations of this study, these findings demonstrated, for the first time, that NDDs employed in CBPs possibly need a more extended rest period and a reassessment of the NDD by the handler after 30 successive anti-drug operations to avoid loss of performance in drug detection. Reasons for the loss of NDDs’ performance could not be determined, but in a real scenario of anti-drug operations such as in this study, we may suggest the need for a greater number of NDDs in anti-drug units to keep a high performance in continuous operations of illicit drug detection in CBPs. A proper determination of a possible more extended rest period and a reassessment of the NDD by the handler after 30 anti-drug operations is still lacking, and further studies need to be carried out.

Analyzing the data showed that the number of illicit drug seizures and success rate in drug detection were influenced by the individual performance of NDDs, with a high variation between dogs (Supplementary Table 3). Assessing dog performance is one of the biggest challenges to overcome in the training and employment of NDDs (16). Considering the wide range in the performance between NDDs employed in CBPs demonstrated in this study, rigorous selection and suitable training programs are crucial to keeping NDDs at appropriate levels for drug detection.

In narcotic detection dog training, NDDs are conditioned to detect multiple chemical components that lead them to indicate a volatile drug-associated odor, which may be different from the original drug (14, 34). An evolution in the narcotics-detecting training process aiming for high-performance NDDs (16) and improvement in the choice and preparation of odorants (scent) (35) are examples of reliable practices to improve NDD detector units. Advances in the training and selection process of NDDs, including odor panel search and accurate simulations in vehicles, luggage, containers, and others, have been proposed to refine NDDs’ performance (16).

Analyzing the data showed that NDDs from German Shepherd and Belgian Shepherd Malinois breeds evaluated are frequently employed in NDD units in Brazil, but no significant differences in behavior traits were experimentally observed between detector and pet dogs (36), suggesting a proper selection and training processes are more important than dog’s breed for high performance in substances detection. Therefore, more studies assessing NDDs in real working scenarios are needed to maintain the performance and systematically adjust the dynamics and levels of narcotic detection according to demand.

Seizures of illicit drugs by NDDs were influenced by the CBPs in this study, with a vast range in type of drug seizures, amount, and frequency of drug apprehensions between CBPs (Supplementary Tables 4, 5). Many factors could affect our results, but the dynamics and routes of trafficking, CBP location, illicit drug demand, border size with drug producers, and flow levels of people, cargo, packages, and luggage in different CBPs are crucial to be considered. CBPs located in airports, ports, post offices, customs, and borders with other countries showed significant differences in the number and type of illegal drug apprehensions by NDDs, which were achievably related to specificities of trafficking, distinct types of drugs, and demand.

Customs Border Posts at the border with Paraguay, the largest producer of marijuana in South America, overcome by violent criminal organizations (37, 38), possibly justify increased seizures of marijuana by NDDs at these locations. Maritime transport is one of the most prevalent ways of cocaine shipment to Europe from South America (8, 39), and inspection of containers and cargo terminals with NDDS in CBPs located at Brazilian ports significantly contributed to large quantities of cocaine seized observed in this study. In CBPs with less equipment and infrastructure to carry out drug detection (staff, scanners, and others) assessed in this study, such as central post offices and smaller airports, NDDs were more critical and strategic for hindering drug trafficking, being able to detect even small portions of illicit drugs in postal correspondences, packages, luggage, and others. In contrast, NDDs had a proportional minor impact on overall narcotics seizures in CBPs located at large and well-structured ports and airport terminals, which has a greater possibility of drug detection by other tools of inspection.

## 5 Conclusion

This article analyzed data from a real scenario of anti-narcotics operations with NDDs in CBPs in Brazil. NDDs had a significant impact on apprehensions of illegal drugs in port and airport terminals, international borders, customs, central post offices, and others, substantially increasing the quantities of drugs seized by every NDD employed. Training protocols and selection of NDDs employed in anti-drug operations enable the detection of a range of different illegal drugs, especially cocaine and derivate products, marijuana and THC-related drugs, and synthetics in Brazil. Significant variations between individual NDDs’ performance reveal the need for constant monitoring and adjustments in the selection, training, and deployment of NDDs in CBPs. In the conditions of this study, we suggest the need for a possible more extended rest period and a reassessment of the NDD by the handler when NDDs reach 30 consecutive anti-drug operations. We also suggest increasing the number of NDDs in CBPs to keep the levels and number of anti-drug operations and performance of dogs. Substantial variations in drug apprehensions and types of drugs seized between distinct CBPs revealed a necessity for more investments in infrastructure, trained people and NDDs, and continued settings and improvement in illegal drugs detection and drug-trafficking hindering.

## Data availability statement

The original contributions presented in the study are included in the article/Supplementary material, further inquiries can be directed to the corresponding author.

## Ethics statement

Approval of this study by an Ethics Committee for Animal Experimentation is not applicable. In this article, we exclusively used aggregated data set and information collected in a real scenario of anti-drug operations on drug seizures, detection dogs, and Customs Border Posts. The Brazilian Customs provided all aggregated data set, and their use was explicitly authorized. Brazilian Customs owned all evaluated detection dogs, which were not submitted to any experimental procedures or protocols previously outlined for this study. All procedures, training, and keeping detection dogs in Brazilian Customs follow the Animal Health and Welfare Act Number 15, 2013, revised and updated to 22 January 2022.

## Author contributions

GJ: Conceptualization, Data curation, Investigation, Methodology, Writing – original draft, Writing – review & editing. CX: Supervision, Writing – original draft, Writing – review & editing, Data curation. MM: Supervision, Writing – original draft, Writing – review & editing, Data curation. MC: Formal analysis, Methodology, Writing – original draft, Writing – review & editing. CoM: Formal analysis, Methodology, Software, Validation, Writing – original draft, Writing – review & editing. CrM: Conceptualization, Funding acquisition, Investigation, Methodology, Resources, Supervision, Writing – original draft, Writing – review & editing.

## Footnotes

^1^Cannabinoids are made in small crystal formations around cannabis flowers, called glandular trichomes.

^2^Skunk often only contains traces of cannabidiol CBD, while hash tends to have roughly equivalent CBD and THC.

## References

[1] Jendrny P Twele F Meller S. Canine olfactory detection and its relevance to medical detection. BMC Infect Dis. (2021) 21:838–15. doi: 10.1186/s12879-021-06523-8, PMID: 34412582 PMC8375464

[2] Riezzo I Neri M Rendine M Bellifemina A Cantatore S Fiore C. Cadaver dogs: unscientific myth or reliable biological devices? Forensic Sci Int. (2014) 244:213–21. doi: 10.1016/j.forsciint.2014.08.026 25264919

[3] DeGreeff LE Peranich K. Canine olfactory detection of trained explosive and narcotic odors in mixtures using a mixed odor delivery device. Forensic Sci Int. (2021) 329:111059. doi: 10.1016/j.forsciint.2021.111059, PMID: 34715445

[4] Uvarova I. A. (2021). “The main directions and forms of international cooperation in the field of illicit drug trafficking” in *Sustainable Development: Society, Ecology, Economy*. Earth and Environmental Sciences Library, Springer, Cham.

[5] McGrath J Saha S Welham J El Saadi O MacCauley C Chant D. A systematic review of the incidence of schizophrenia: the distribution of rates and the influence of sex, urbanicity, migrant status and methodology. BMC Med. (2004) 2:13. doi: 10.1186/1741-7015-2-13, PMID: 15115547 PMC421751

[6] Di Forti M Quattrone D Freeman TP Tripoli G Gayer-Anderson C Quigley H. The contribution of use to variation in the incidence of psychotic disorder across Europe (EU-GEI): a multicentre case-control study. Lancet Psychiatry. (2019) 6:427–36. doi: 10.1016/S2215-0366(19)30048-3, PMID: 30902669 PMC7646282

[7] Espejo MP. Drug-trafficking in Colombia: the new civil war against democracy and peacebuilding. Co-herencia. (2021) 18:157–92. doi: 10.17230/co-herencia.18.34.6

[8] Aziani A Berlusconi G Giommoni L. A quantitative application of Enterprise and social embeddedness theories to the transnational trafficking of cocaine in Europe. Deviant Behav. (2021) 42:245–67. doi: 10.1080/01639625.2019.1666606

[9] Williams P. Transnational criminal networks In: Arquilla J Ronfeldt D, editors. Networks and Netwars: The Future of Terror, Crime and Militancy. Washington DC: RAND Corporation (2001)

[10] Moser AY Bizo L Brown WY. Olfactory generalisation in detector dogs. Animals. (2019) 9:702. doi: 10.3390/ani9090702, PMID: 31546835 PMC6769875

[11] Micheletti MH de Paula AC de Sá MEP de Melo CB. Detection dogs: a brief review about the use of the canine nose. Braz J Vet Med. (2016) 38:387–92.

[12] Otto CM Guest CM. Canine Olfactory Detection. Lausanne: Frontiers Media SA (2020). 10.3389/fvets.2020.00100 PMC7056699 32175340

[13] Francis VS Holness HK Furton KG. The ability of narcotic detection canines to detect illegal synthetic Cathinones (Bath salts). Front Vet Sci. (2019) 6:98. doi: 10.3389/fvets.2019.00098, PMID: 31024937 PMC6465326

[14] Furton KG Hong Y Hsu L Luo T Rose S Walton J. Odor signatures of cocaine analysed by GC/MS and threshold levels of detection for drug detection canines. J Chromatogr Sci. (2002) 40:147–55. doi: 10.1093/chromsci/40.3.147, PMID: 11954652

[15] Lorenzo N Wan TL Harper RJ Hsu Y Chow M Rose S. Laboratory and field experiments used to identify *Canis lupus* var. familiaris active odor signature chemicals from drugs, explosives, and humans. Anal Bioanal Chem. (2003) 376:1212–24. doi: 10.1007/s00216-003-2018-7, PMID: 12845400

[16] Jantorno GM Xavier CH de Melo CB. Narcotic detection dogs: an overview of high-performance animals. Ciência Rural. (2020) 50:10. doi: 10.1590/0103-8478cr20191010

[17] Jezierski T Adamkiewicz E Walczak M Sobczynska M Bruzda AG Ensminger J. Efficacy of drug detection by fully-trained police dogs varies by breed, training level, type of drug and search environment. Forensic Sci Int. (2014) 237:112–8. doi: 10.1016/j.forsciint.2014.01.013, PMID: 24631776

[18] Sindireceita (2019). Programa K9 da Receita Federal do Brasil. Available at: https://sindireceita.org.br/images/bkp/uploads/2018/12/cartilha-k9-web.pdf

[19] Ministério da Economia, Brasil (2020). Receita Federal aprende mais de 57 toneladas de cocaína em 2019. Available at: https://www.gov.br/pt-br/noticias/justica-e-seguranca/2020/01/receita-federal-apreende-mais-de-57-toneladas-de-cocaina-em-2019

[20] SAS Institute Inc. (2023). SAS Campus Drive, Cary, North Carolina 27513, USA.

[21] UNODC (2022). “Dogs from the anti-narcotics police dog training center responsible for 15% of total drug seizures in Iran” in United Nations Office on Drugs and Crime. Available at: https://www.unodc.org/islamicrepublicofiran/en/dogs-from-the-anti-narcotics-police-dog-training-center-responsible-for-15percent-of-total-drug-seizures-in-iran.html

[22] INCSR (2021). Drug and chemical control. Volume I, march 2021. United States Department of State Bureau of international narcotics and law enforcement affairs. International Narcotics Control Strategy Report. Available at: https://www.state.gov/2021-international-narcotics-control-strategy-report/

[23] UNODC (2019). “Wholesales drug price in US$. Wholesale drug price and purity level: 2019” in United Nations Office on Drugs and Crime. Available at: https://dataunodc.un.org/data/drugs/Wholesale%20drug%20price

[24] UNODC (2021). “Price and purity” in United Nations Office on drugs and crime. Available at: https://www.unodc.org/documents/data-and-analysis/WDR2021/8.1_Prices_an_purities_of_Drugs.pdf

[25] Mason A Sami M Notley C Bhattacharyya S. Are researchers getting the terms used to denote different types of recreational cannabis right?—a user perspective. J Cannab Res. (2021) 3:1–10. doi: 10.1186/s42238-021-00065-1, PMID: 33926566 PMC8086348

[26] RINCB (2023). International Narcotics Control Board. International Narcotics Control Board for 2022 (E/INCB/2022/1). Report 2022. Vienna, Austria. Available at: https://www.incb.org/incb/en/publications/annual-reports/annual-report-2022.html

[27] Liu Y Richards VL Gebru NM Spencer EC Cook RL. Associations amongst form of cocaine used (powder vs crack vs both) and HIV-related outcomes. Addict Behav Rep. (2021) 14:100374. doi: 10.1016/j.abrep.2021.100374, PMID: 34938835 PMC8664776

[28] Khullar V Jain A Sattari M. Emergence of new classes of recreational drugs—synthetic cannabinoids and Cathinones. J Gen Intern Med. (2014) 29:1200–4. doi: 10.1007/s11606-014-2802-4, PMID: 24553958 PMC4099455

[29] UNODC (2017). “Global synthetic drugs assessment. Amphetamine-type stimulants and new psychoactive substances” in United Nations Office on drugs and crime (2017). Vienna: United Nations. Available at: https://www.unodc.org/documents/scientific/Global_Drugs_Assessment_2017.pdf

[30] Pardal M Colman C Surmont T. Synthetic drug production in Belgium–environmental harms as collateral damage? J Illicit Econom Dev. (2021) 3:36–49. doi: 10.31389/jied.84

[31] Romano F Tomazzoni EL Uvinha RR. Mega-events in Brazil and the National Tourism Plan 2013-2016: the goals of tourist expansion. Rev Rosa Ventos Turismo Hospital. (2019) 11:454–75. doi: 10.18226/21789061.v11i2p454

[32] UNODC (2022a). World drug report. Analysis of drug markets. Drug market trends cannabis opioids in United Nations Office on drugs United Nations publications, Austria. Available at: https://www.unodc.org/res/wdr2022/MS/WDR22_Booklet_3.pdf

[33] Magliocca NR Summers DS Curtin KM McSweeney K Price AN. Shifting landscape suitability for cocaine trafficking through Central America in response to counterdrug interdiction. Landscape Sci Plan. (2022) 221:104359. doi: 10.1016/j.landurbplan.2022.104359

[34] Macias M. S. Harper R. J. Furton K. G. (2008). A comparison of real versus simulated contraband VOCs for reliable detector dog training utilising SPME-GC-MS. Am Lab. Available at: https://www.americanlaboratory.com/913-Technical-Articles/707-A-Comparison-of-Real-Versus-Simulated-Contraband-VOCs-for-Reliable-Detector-Dog-Training-Utilizing-SPME-GC-MS/

[35] Hayes JE McGreevy PD Forbesc SL Laing G Stuetz RM. Critical review of dog detection and the influences of physiology, training, and analytical methodologies. Talanta. (2018) 185:499–512. doi: 10.1016/j.talanta.2018.04.010, PMID: 29759233

[36] Hare E Kelsey KM Serpell JA Otto CM. Behavior differences between search-and-rescue and pet dogs. Front Vet Sci. (2018) 5:118. doi: 10.3389/fvets.2018.00118, PMID: 29922685 PMC5996094

[37] Moriconi M Peris CA. Merging legality with illegality in Paraguay: the cluster of order in Pedro Juan Caballero. Third World Q. (2019) 40:2210–27. doi: 10.1080/01436597.2019.1636225

[38] Moriconi M Peris CA. Cultivating Cannabis in a paraguayan nature reserve: incentives and moral justification for breaking the law. Trends Organ Crime. (2022). doi: 10.1007/s12117-022-09464-z

[39] UNODC (2022b). World drug report. “Drug market trends cocaine amphetaminetype stimulants new psychoactive substances” in *United Nations Office on Drugs United Nations Publications, Austria*. Available at: https://www.unodc.org/res/wdr2022/MS/WDR22_Booklet_4.pdf

