# Supplementary material for: Detection dogs fighting transnational narcotraffic: performance and challenges under real customs scenario in Brazil
Source: Front Vet Sci. 2024 May 16;11:1380415. doi: 10.3389/fvets.2024.1380415 (PMC11137163; doi:10.3389/fvets.2024.1380415)
Supplement: Supplementary file 1 [file Data_Sheet_1.pdf]

Detection dogs fighting transnational narcotraffic: performance and challenges under real Customs scenario in Brazil

Gustavo Machado Jantorno [1,2], Carlos Henrique Xavier [2,3], Marcelo Eduardo Peixoto Magalhães [2], Márcio Botelho de Castro [1], Concepta McManus [1], Cristiano Barros de Melo [1, *]

[1] Graduate Program in Animal Sciences (PPGCA/FAV), University of Brasilia (UnB), Campus Darcy Ribeiro, ICC Sul, Asa Norte, Brasilia, Federal District, Brazil
[2] Center for Detection Dogs (CNK9), Customs/Aduana, Vitória, Espirito Santo, Brazil
[3] Head of the Center for Detection Dogs (CNK9), Customs/Aduana, Vitória, Espirito Santo, Brazil

Supplementary Material

Supplementary Material - Table 1. Narcotics seizures (kg/year) detected by NDDs in Brazilian CBPs (2010 to 2020).

Year	2010	2011	2012	2013	2014	2015	2016	2017	2018	2019	2020
**Marijuana**	0	400	936	925	3039	1,359	944	713	980	1,530	1,598
**Crack**	0	11	86	13	48	27	23	2	0	16	2
**Pasta**	0	0	0	269	530	706	0	1	40	565	28
**Cocaine**	49	25	270	166	133	699	7,012	7,763	19,563	14,520	16,220
**Hashish**	0	6	16	19	36	729	44	65	42	130	669
**Ecstasy[1]**	0	0	2,500	16,244	49,012	20,523	14,362	96,594	8,796	61,964	33,957
**LSD[2]**	0	0	0	0	0	12,825	76,354	1,017	2,112	2,792	19,519
**Meth**	0	0	0	0	0	2	1	59	47	13	1
**Heroin**	0	0	0	0	0	0	0	0	60	0	0
**MDMA[3]**	0	0	0	0	0	0	50	4	15	35	1
**Skunk[4]**	0	0	0	0	0	0	0	436	226	3,300	3,945

Source: Sindireceita 2019 (**18**); Brasil 2020 (**19**). [1] Usually sold as tablets; [2] Lysergic acid diethylamide- usually sold as blotters; [3]n-methyl-3,4-methylenedioxymethamphetamine; [4] skunk is a form of marijuana.

Supplementary Material - Table 2. Influence of the dog and number of operations in the number of seizures and in the month.

DOG (NDD)	N	MEAN
K9-23	6	10.833 a
K9-19	6	8.833 a,b
K9-24	6	7.333 a,b
K9-9	6	5.500 b,c
K9-7	6	4.833 b,c,d
K9-26	6	3.167 c,d,e
K9-10	6	2.167 c,d,e
K9-21	6	1.833 c,d,e
K9-27	6	1.667 c,d,e
K9-15	6	1.167 d,e
K9-22	6	0.833 d,e
K9-11	6	0.833 d,e
K9-12	6	0.667 e
K9-13	6	0.333 e
K9-1	6	0.333 e
K9-18	6	0.167 e
K9-16	6	0.167 e
K9-17	6	0.167 e
K9-14	6	0.000 e
K9-25	6	0.000 e
K9-2	6	0.000 e
K9-4	6	0.000 e
K9-20	6	0.000 e
K9-6	6	0.000 e
K9-3	6	0.000 e
K9-8	6	0.000 e
K9-5	6	0.000 e

Tukey test ($P<0.05$). Means with the same letter are not significantly different. N = amount of months/repetition. NDD = Narcotic Detection Dog. The NDD name identification and other dog specifications have been protected for security reasons.

Supplementary Material - Table 3. Comparison for Success Rate, among dog (NDDs), number of operations and month.

DOG (NDD)	N	MEAN
K9-23	6	60.837 a
K9-27	3	52.778 a,b
K9-9	5	50.866 a,b
K9-24	6	49.028 a,b,c
K9-7	5	43.556 a,b,c,d
K9-19	6	33.490 a,b,c,d,e
K9-26	6	27.679 a,b,c,d,e
K9-10	6	20.462 b,c,d,e
K9-11	6	14.762 c,d,e
K9-12	6	12.381 d,e
K9-21	4	10.962 d,e
K9-1	6	10.714 d,e
K9-22	6	9.307 d,e
K9-13	6	7.500 e
K9-15	6	2.768 e
K9-18	6	1.852 e
K9-16	6	1.389 e
K9-17	6	1.389 e
K9-20	5	0.000 e
K9-3	4	0.000 e
K9-5	6	0.000 e
K9-4	3	0.000 e
K9-8	6	0.000 e
K9-25	5	0.000 e

Tukey test ($P<0.05$). Means with the same letter are not significantly different. N = amount of months/repetition. NDD = Narcotic Detection Dog. The NDD name identification and other dog specifications have been protected for security reasons.

Supplementary Material - Table 4. Tukey test analysis on the influence of the number of operations and CBP in the seizure of narcotics seizure by NDDs.

CBP	N	MEAN
CBP-5	6	18.167 a
CBP-3	6	12.000 b
CBP-7	6	8.833 b,c
CBP-9	6	3.167 c,d
CBP-8	4	2.750 d
CBP-15	6	2.167 d
CBP-4	6	1.833 d
CBP-2	6	1.167 d
CBP-12	6	0.833 d
CBP-1	6	0.500 d
CBP-13	6	0.333 d
CBP-11	5	0.000 d
CBP-6	6	0.000 d
CBP-10	5	0.000 d
CBP-16	4	0.000 d
CBP-17	2	0.000 d

Tukey test ($P<0.05$). Means with the same letter are not significantly different. N = amount of months/repetition. CBP – Customs Border Post. The border name identification and other border specifications have been protected for security reasons.

Supplementary Material - Table 5. Comparison between Success Rate (%) of CBP in the seizure of narcotics seizure (Tukey test).

CBP	N	MEAN
CBP-5	6	55.630 a
CBP-7	6	33.488 a,b
CBP-9	6	27.678 a,b,c
CBP-3	6	24.948 b,c
CBP-15	6	20.462 b,c
CBP-4	6	11.707 b,c
CBP-8	4	10.963 b,c
CBP-13	6	10.715 b,c
CBP-12	6	9.307 b,c
CBP-2	6	2.768 c
CBP-1	6	1.515 c
CBP-11	5	0.000 c
CBP-6	6	0.000 c
CBP-10	5	0.000 c
CBP-16	4	0.000 c
CBP-17	2	0.000 c

Tukey test ($P<0.05$). Means with the same letter are not significantly different. **N** = amount of months/repetition. **CBP** = Custom Border Post. The border name identification and other border specifications have been protected for security reasons.

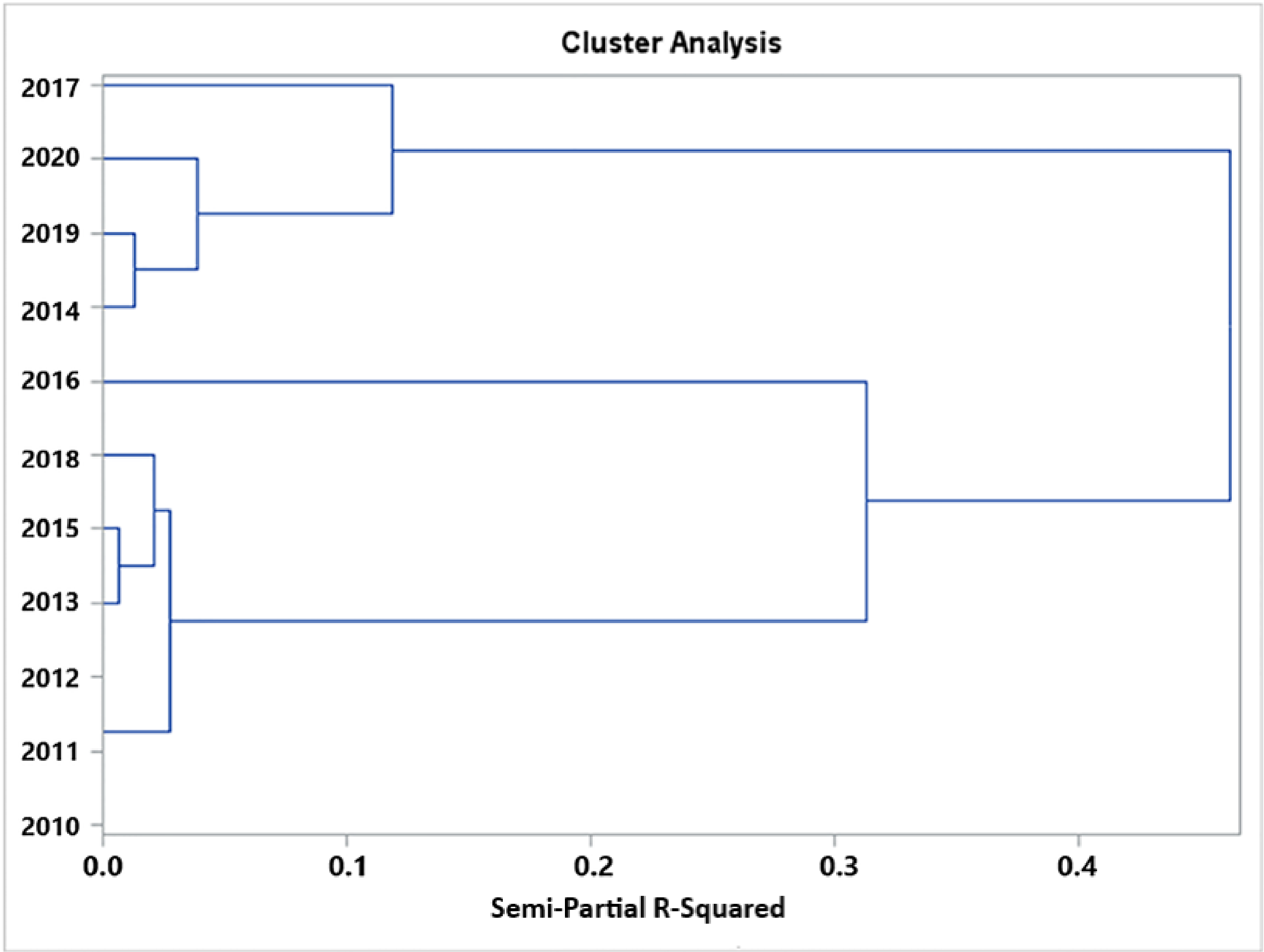

Supplementary Material - Figure 1. Cluster analysis of years (2010 to 2020) considering drug seizures detected by NDDs (K9 units).

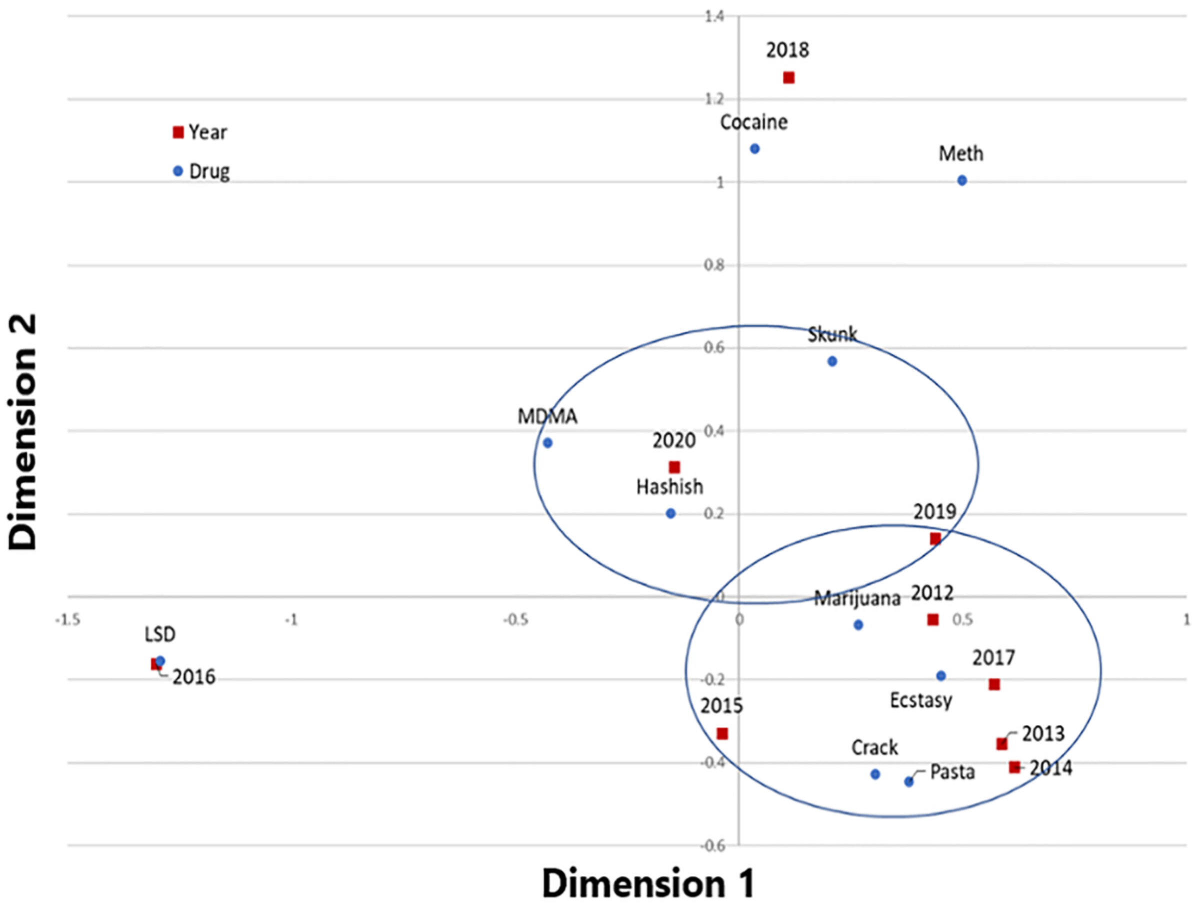

Supplementary Material - Figure 2. Correspondence Analysis between years (2012 to 2020) and narcotic seizures by NDDs in Brazil. MDMA = 3,4-Methylenedioxymethamphetamine; Meth = Methamphetamine; Pasta = Basic cocaine paste; LSD = Lysergic Acid Diethylamide.

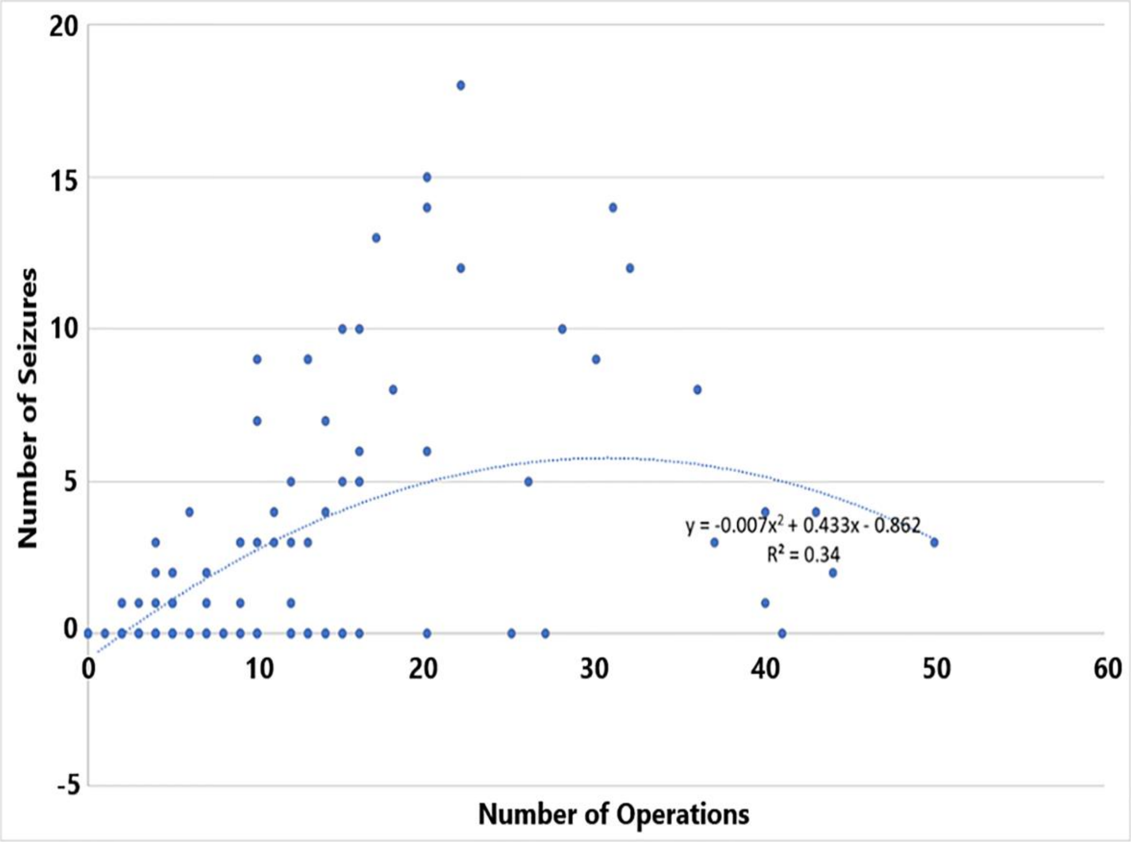

Supplementary Material - Figure 3. Polynomial regression analysis of the number of anti-drug operations using NDDs and the number of seizures for narcotics detection.
